# PFAAs in Fish and Other Seafood Products from Icelandic Waters

**DOI:** 10.1155/2014/573607

**Published:** 2014-03-20

**Authors:** Hrönn Jörundsdóttir, Thorhallur I. Halldorsson, Helga Gunnlaugsdottir

**Affiliations:** ^1^Matis, Icelandic Food and Biotech R&D, Vinlandsleid 12, 113 Reykjavik, Iceland; ^2^Faculty of Food Science & Nutrition, School of Health Science, University of Iceland, Unit of Nutritional Research, Eiríksgötu 29, 101 Reykjavik, Iceland

## Abstract

Perfluorinatedalkyl acids (PFAAs) are of growing concern due to possible health effects on humans. Exposure assessments indicate that fish consumption is one of the major sources of perfluorooctane sulfonate (PFOS) exposure to humans, one of the major PFASs, whereas concerns of overestimation of this exposure source have been raised. Therefore, PFAAs concentrations in fish from the North Atlantic (Icelandic fishing grounds) in the flesh of different fish species were investigated along with more detailed analyses of tissue concentrations in cod (*Gadus morhua*) and lumpfish (*Cyclopterus lumpus*). Further, fish feed was investigated as a possible source of PFAAs in aquaculture by examining fish meal as feed ingredient. No PFAAs were detected in the edible part of all fish samples, except for PFOS in pollock (*Pollachius virens*, 0,05 ng/g wet weight). PFOS was the only PFAA detected in the fish meal samples with the exception of PFOSA in blue whiting (*Micromesistius poutassou*) meal (0,45 ng/g dry weight (d.w.)), where the PFOS concentration was 1,3–13 ng/g d.w. in the capelin (*Mallotus villosus*) and mackerel (*Scomber scombrus*) meal samples. The conclusions of the study are that fish commonly consumed from the Icelandic fishing grounds are unlikely to be an important source of PFAAs exposure.

## 1. Introduction

Perfluorinated alkyl acids (PFAAs) are a group of highly persistent compounds consisting of fully fluorinated carbon chain (C4–C14) with an active terminal group, main emphasis in the present study will be on compounds having either a terminal carboxylate or sulfonate. These compounds are not lipophilic but have protein affinity and are ubiquitous in the environment as reviewed by Butt et al., 2010 [1]. These compounds, or their precursors, have been industrially produced for the last 50 years and are used widely in the industry with varying application due to their unique chemical and physical properties [2]. In 2000, the largest producer worldwide of products based on perfluorooctanesulfonyl fluoride (POSF) chemistry, 3 M, announced that these products would voluntarily be phased out [3]. However, fluorotelomer alcohols which can degrade to perfluorocarboxylic acids (PFCAs) [4] and other homologues with various chain lengths are still produced [5] and PFOS is still produced in China [6]. PFAAs with longer chain length (>C7 for PFCAs and >C5 for perfluorinated alkylsulfonic acids (PFSAs)) are considered to be bioaccumulative [7] and the PFAA commonly found in the highest concentration in biota, perfluorooctane sulfonate (PFOS) [1], has been added to the Stockholm convention of persistent organic pollutants (POPs). Furthermore, the marketing and use of PFOS have been restricted within EU according to Directive 2006/122/EC. After the phaseout of PFOS and PFOS related compounds, the environmental concentration of PFOS started to decrease in some matrixes but not in polar bear liver from East Greenland [8, 9].

Food and food packaging and water and house dust are suggested to be major sources for humans exposure as reviewed in several studies even if the relative importance of each source is still unknown [10–13]. Comparing different food groups, food of marine origin has been identified as an important source of PFOS to humans but is probably very dependent on catch area [14–16]. Therefore, depending on study area, this particular exposure route might be overestimated, as the data available might be influenced by studies from relatively polluted areas and there is a need for studies on commonly consumed seafood [17]. It is therefore important to assess the concentration of PFAAs in different seafood and seafood products as well as to investigate all sources of PFAAs into the food chain, such as with feed.

The aim of the project was to investigate PFAA concentration in different seafood products from Icelandic waters, both products for human consumption as well as products used as feed due to the bioaccumulative and biomagnifying properties of several PFAA homologues [18–20], which could lead to increased concentrations of these substances up the food chain. Six perfluorocarboxylic acids (PFCAs), four perfluoroalkane sulfonates, and perfluorooctane sulfonamide (PFOSA) were analysed. The samples investigated were taken as part of the annual Icelandic surveillance project conducted by Matis Ltd. (Icelandic Food and Biotech R&D) on behalf of the Ministry of Industries and Innovation. This project involves screening of different organic and inorganic contaminants in seafood and seafood products. In general, limited data is available on PFAAs in the Icelandic environment, food commodities, and population [21, 22].

## 2. Material and Methods

### 2.1. Sampling and Pretreatment

Atlantic cod (*Gadus morhua*), lumpfish (*Cyclopterus lumpus*), pollock (*Pollachius virens*), ling (*Molva molva*), plaice (*Pleuronectes platessa*), lemon sole (*Pseudopleuronectes americanus*), anglerfish (*Lophius piscatorius*), and blue whiting (*Micromesistius poutassou*) samples were collected by the Icelandic Marine Research Institute in March, 2011, during their biannual scientific surveys. Cod and anglerfish were caught south-west of Iceland, blue whiting was caught south-east of Iceland, and lumpfish and pollock were caught north-west of Iceland, while ling, plaice, and lemon sole were caught west of Iceland. The fish was drained of blood (bled) immediately after it was taken on board according to common practise within the fishing industry and then frozen whole, that is, ungutted. No further sample preparation was performed on board the fishing vessel. The frozen fish was then transported to the laboratory, thawed at room temperature, gutted, and filleted at the laboratory where prevention of any cross-contamination was emphasised. No blanks were prepared, neither at the vessel nor at the laboratory. As the samples were analysed for further compounds than the PFAAs, all tools were cleaned with distilled water and 2% sodium citrate and EDTA solution and all glassware was cleaned with concentrated nitric acid (HNO_3_) and heated at 300°C overnight. In total, 13 different fish samples (flesh) and internal/external organs from six of these samples were investigated. Each fish sample consisted of a pooled sample from the entire edible part (e.g., skinless fish fillet) from at least ten individuals of the same species within a specific size range (please refer to Table 1 for further details). For the internal organs, the livers consisted of a pooled sample from 10 individuals, while the number of roe and sperm in the pooled cod samples depended on the ratio of males and females. All lumpfish samples were female. Cod sample 1 consisted of five males and five females, while cod sample 2 consisted of eight males and two females. Unfortunately, as the samples were originally intended for routine surveillance of environmental pollution and food safety, no internal organs were collected from other fish species. Samples and organs collected are presented in Table 1. Samples were homogenized and kept frozen (−20°C) until transport to contract laboratory abroad.

Five capelin (*Mallotus villosus*) meals and corresponding oil samples as well as one mackerel (*Scomber scombrus*) meal sample and one blue whiting meal sample were gathered from commercial industrial producers in Iceland. Emphasis was laid on obtaining fishmeal and fish oil from the same production lot to be able to compare distribution of PFAAs between meal and oil. Samples were taken by commercial industrial producers directly from the end products at the production site and send to Matis. Fish meal samples were stored at room temperature and oil samples frozen (−20°C) until transport to contract laboratory abroad.

All samples were sent frozen to Eurofins (Hamburg, Germany) for chemical analyses with courier transport. The contract laboratory in Hamburg confirmed that the samples were still frozen upon arrival.

### 2.2. Chemical Analyses and Quality Assurance

All samples were analysed by Eurofins WEJ Contaminants Laboratory, Hamburg, Germany, which is accredited according to the international standard DIN EN ISO/IEC 17025:2005 for PFAAs. The accreditation includes an extensive quality system including blanks, traceability, accuracy, precision, and participation in interlaboratory comparison (ring tests) in order to ensure result quality as well as analyses reference material and blank samples simultaneously with samples. ^13^C isotope labelled surrogate standards (^13^C_4_-PFBA, ^13^C_2_-PFHxA, ^13^C_8_-PFOA, ^13^C_5_-PFNA, ^13^C_2_-PFDA, ^13^C_2_-PFUnA, and ^13^C_4_-PFOS) are added to the homogenized sample, and analytes are then extracted with methanol. The sample is cleaned with SPE (STRATA-X-AQ) and recovery standard (^13^C_4_-PFOA) was added prior to analysis with a liquid chromatograph coupled with a tandem mass spectrometer (LC-MS/MS). Recovery standard is added just prior to LC/MS/MS analyses. PFAA compounds analyses were perfluorohexanoate (PFHxA), perfluoroheptanoate (PFHpA), perfluorooctanoate (PFOA), perfluorononanoate (PFNA), perfluorodecanoate (PFDA), perfluorododecanoate (PFDoA), perfluorobutane sulfonate (PFBS), perfluorohexane sulfonate (PFHxS), perfluorooctane sulfonate (PFOS), perfluorodecane sulfonate (PFDS), and perfluorooctane sulfonamide (PFOSA).

The fat content of the samples was analysed according to AOCS [23].

## 3. Results

Results and LODs of fish flesh and other organs are presented in Table 1 and results and LODs for fish meal and fish oil are presented in Table 2. No PFAA was detected above limits of detection in any flesh sample except for PFOS in pollock, 0,05 ng/g w.w. No PFAA was detected in the different lumpfish organs investigated. In the cod organs investigated from the two pools investigated, only PFOS was detected, with highest concentration in roe (2,7 and 2,8 ng/g, resp.), follow by either sperm or liver, depending on pool, ranging from 0,27 to 0,62 ng/g w.w. No PFAA was detected above limits of detection (LOD) in fish oil samples where PFOS was the only PFAA detected above LOD in the fish meal samples ranging between 1,3 and 13 ng/g, with the exception of blue whiting meal, where PFOSA was detected (0,45 ng/g) but not PFOS.

## 4. Discussion

### 4.1. PFOS Tissue Distribution in Fish

No PFAAs were detected in most fish flesh samples except for pollock where PFOS was detected (0,05 ng/g wet weight (w.w.), Table 1). In other fish organs, PFOS was detected in liver, roe, and sperm from cod but not in the cod flesh (Table 1) with the detection limit of 0,15 ng/g w.w. PFOS is generally in highest concentration in blood, followed by kidney, liver, brain, and muscle in fish [19, 24]. Berger et al. report higher levels of PFOS, between 0,47 and 23,1 ng/g w.w. in fresh and brackish water fish flesh from Sweden [14] where PFOS is reported in levels between 0,3 and 2,9 ng/g w.w. in Arctic cod flesh [25, 26] compared to the present study where PFOS was below LOD. Nevertheless, none of the studies mentioned above state whether the fish bled after it was caught as was done in the present study. If the fish was not allowed to bleed prior to filleting, this could increase the PFOS concentration in the flesh. The levels of PFOS in fish blood are most likely low in the present study as the concentration was low in the liver, which has higher tissue concentration as shown by Mortesen et al. [27]. Therefore, whether the bleeding is the reason for the discrepancy between the present study and previous studies or whether fish from the Icelandic fishing area is less exposed to PFAAs compared to fish from other marine areas must be investigated further. In the present study, the blood was removed from the fish on-board the fishing vessel as is done for fish caught for human consumption and not analysed; the largest portion of the PFOS has thus probably been removed from the flesh. However, immediate removal of the blood is standard for all commercial fish intended for human consumption and tissue concentrations after bleeding therefore give a better estimation of the exposure to PFOAS via fish as consumed. The results of the present study indicate that consumption of North Atlantic fish is unlikely to be an important source of PFOS or other PFAAs to humans.

Table 1 shows the tissue distribution of PFOS in three other cod organs apart from flesh, that is, roe, sperm, and liver. PFOS concentration is highest in roe compared to sperm and liver. In the two different cod pools, the roe/liver ratio is 4,3 and 8,1, respectively (Table 1). This ratio is higher compared to the roe/liver ratio ranging between 0,93 and 2,0 found in farmed fresh water fish [24]. In the farmed fresh water fish, the roe concentration ranged between 1,64 and 5,07 ng/g w.w. compared to the PFOS concentration of 2,7 and 2,8 ng/g w.w. in the cod roe in the present study. The roe/liver ratio has been used to evaluate maternal transfer [24, 28]. The roe/liver ratio in cod in the present study indicates efficient maternal transfer to offspring in cod and the difference in this ratio compared to the ratio presented by Shi et al. indicates that this transfer may differ between fish species [24]. Tissue distribution in the fresh water fish species carp and snakehead from Asia revealed liver/muscle ratio of PFOS concentration ranging between 3,1 and 52 with a considerable variation within both species [24]. The liver PFOS concentration ranged between 3,7 and 69 ng/g w.w. in carp and between <0,5 and 12 in snakehead and the results also indicate a possible relation between liver concentration and liver/muscle ratio [29]. The PFOS concentrations in cod livers in the present study are in the lower range of the concentrations detected by Murakami et al. [29]. The cod samples analysed here were from remote Arctic waters while the carps analysed by Murakami et al. were caught close to sewage treatment plant outflow in Tokyo and two out of six carps showed reproductive anomalies [29]. These samples are therefore clearly from different exposure level, since there is no known local source of PFAAs in the North Atlantic.

Roes are eaten by certain subpopulations in Iceland, especially the older generation. Given the assumption of an average 250 g portion of roe and the average roe concentration of 2,8 ng/g, this gives a 700 ng dose of PFOS. EFSA recommends a 150 ng/kg/day tolerable daily intake of PFOS which would result in a 10.500 ng/day for an average 70 kg person [17, 30]. One portion of cod roe would therefore supply a person of the large part of the tolerable daily intake of PFOS where other sources have not been accounted for. Nevertheless, cod roes are rarely eaten on daily basis even in Icelandic fishing communities.

### 4.2. PFOS in Fish Meal and Fish Oil

PFOS was the only PFAA detected in capelin and mackerel meal (2,3–13 ng/g), whereas PFOSA was the only PFAA detected in blue whiting meal (0,45 ng/g). No PFAAs were detected in the capelin oil samples (Table 2), since PFOSA was not detected in blue whiting flesh, indicating that the PFOSA source is most likely from an internal organ included in the meal. Mean PFOS concentration in capelin meal (*n* = 5) was 7,3 ng/g while the PFOS concentration is lower in the only mackerel meal sample analysed compared to the capelin meal sample, 1,3 ng/g w.w. Average lipid content of the capelin meal is 13% while the lipid content of the mackerel meal is 10,1%, suggesting (as expected) that the lipid content is not an indicator of PFOS concentration. No linear correlation was seen between lipid content in the mackerel samples and the PFOS concentration which is not surprising since PFOS is not lipid bound. The raw material used in these meal types differs considerably. In fact, the raw material can differ between batches within the same meal type and fishing grounds and even season could influence the PFOS concentration. Largest part of mackerel caught around Iceland is used for human consumption, but mackerel that is not fit for human consumption due to low quality as well as cutoffs (heads, internal organs, etc.) from fish after the fillets have been removed is used to produce mackerel meal. Capelin meal is comprised mostly of whole fish where only the roes have been removed from the female. Blue whiting meal on the other hand comprises the entire fish, since no part of the fish is generally used to produce products for human consumption. Some capelin meal batches only comprise male individuals (Thórhallur Jón Jónasson, quality manager of Síldarvinnslan (http://www.svn.is/), personal communication, 2012), which can affect the PFOS concentration in the meal batch. Detailed tissue distribution is necessary in order to be able to predict possible effects of different raw materials in the fish meal production.

Fish meal is a major ingredient used in feeding aquaculture but is also used to some extent in feeding agricultural animals [31, 32]. Fish meal can serve as a source of PFOS to humans through food, that is, aquaculture farmed fish and agriculture farm products due to the biomagnification ability of PFOS [20, 25]. It is therefore important to investigate further PFAA content of fish meal. As shown in the current study, the PFAA concentration and profile are dependent on fish species used in the fish meal. Personal communication with industrial partners reveals that the exact material used in each meal batch can differ, that is, use of whole fish or only parts of the fish. This varies with species, season, quality of the flesh for human consumption, and so forth. No information on seasonal difference of PFAAs in fish meal exists. Further investigations on PFAAs concentration in aquaculture fish are recommended.

## 5. Conclusions

Fish for human consumption contained low or nondetectable concentration of PFAAs, including PFOS and PFOA. Therefore, the results presented in the present study indicate that fish consumption of species caught in the North Atlantic Sea is a limited source of exposure of PFAAs to humans. Large part of the Icelandic fish export is destined for the European market and these results therefore impact the exposure assessment of the European population as well as the Icelandic.

Fish meal contained quantifiable levels of PFOS and PFOSA, probably because the internal organs from the fish species are included in the raw material used for fish meal production. Differences in PFOS levels were observed both between fish meal species as well as within fishmeal batches produced from the same fish species. Fish meal is an important feed ingredient for both aquaculture and agriculture animals; therefore, it is important to investigate further food origination from these sources, especially farmed fish which there is limited information available about.

## Figures and Tables

**Table 1:** Concentration of PFOS in fish organs in ng/g wet weight in each pooled sample. Each pool consists of ten individuals whereas the gender specific organs consist of fewer individuals as stated in the table.

Sample number	Sample name	*N* (♂/♀)	Tissue	Size (cm)	PFOS (ng/g)
1	Cod	10 (5/5)	Flesh	60–74	<0,15
			Roe		2,7
			Sperm		0,27
			Liver		0,62
2	Cod	10 (8/2)	Flesh	75+	<0,15
			Roe		2,8
			Sperm		0,43
			Liver		0,35
3	Lumpfish	10 (0/10)	Flesh	35–45	<0,15
			Roe		<0,15
4	Lumpfish	10 (0/10)	Flesh	39–45	<0,15
			Roe		<0,15
5	Lumpfish	10 (0/10)	Flesh	39–45	<0,15
			Roe		<0,15
			Skin		<0,13
6	Lumpfish	10 (0/10)	Flesh	N.N.	<0,14
			Liver		<0,18
			Skin		<0,15
7	Pollock	10 (N.N)	Flesh	61–69	0,050
8	Ling	10 (N.N)	Flesh	55–81	<0,049
9	Plaice	10 (N.N)	Flesh	30–41	<0,046
10	Lemon sole	10 (N.N)	Flesh	29–39	<0,043
11	Anglerfish	10 (N.N)	Flesh	41–89	<0,049
12	Blue whiting	10 (N.N)	Flesh	33–39	<0,056
13	Blue whiting	10 (N.N)	Flesh	20–30	<0,047

LOD PFHxA, PFHpA, PFOA, PFNA: flesh, roe, sperm: 0,10 ng/g, liver: 0,30 ng/g.

LOD PFDA, PFDoA: flesh, roe, sperm: 0,20 ng/g, liver: 0,30 ng/g.

LOD PFBS, PFHxS, PFDS, PFOSA: flesh, roe, sperm: 0,15 ng/g, liver: 0,30 ng/g.

LOD PFOS: flesh, roe: 0,15 ng/g.

N.N: not known.

**Table 2:** Concentration of PFOS in fish meal/oil for feed in ng/g fresh weight. Concentration in fish meal is calculated for 12% moisture content.

Sample number	Sample name	PFOS (ng/g)	PFOSA (ng/g)	Fat content (%)
14	Capelin meal	6,5	<0,50	12,2
	Capelin oil	<0,50	<0,50	
15	Capelin meal	7,5	<0,50	14,1
	Capelin oil	<0,50	<0,50	
16	Capelin meal	2,3	<0,50	13,5
	Capelin oil	<0,50	<0,50	
17	Capelin meal	13	<0,50	12,4
	Capelin oil	<0,50	<0,50	
18	Capelin meal	6,9	<0,50	13,5
	Capelin oil	<0,50	<0,50	
19	Mackerel meal	1,3	<0,50	10,1
20	Blue whiting meal	<0,24	0,45	9,81

LOD PFHxA, PFHpA, PFOA, PFNA: capelin meal/oil: 0,50 ng/g, mackerel meal: 0,20 ng/g.

LOD PFDA, PFDoA: capelin meal/oil: 0,50 ng/g, mackerel meal: 0,20 ng/g.

LOD PFBS, PFHxS, PFDS, PFOSA: capelin meal/oil: 0,50 ng/g, mackerel meal: 0,20 ng/g.

LOD PFOS: oil: 0,50 ng/g.
